# Self-criticism and self-esteem in early adolescence: Do they predict depression?

**DOI:** 10.1371/journal.pone.0244182

**Published:** 2020-12-18

**Authors:** Catherine B. Gittins, Caroline Hunt

**Affiliations:** 1 School of Psychology, The University of Sydney, Sydney, New South Wales, Australia; 2 College of Health & Human Sciences, Charles Darwin University, Darwin, Northern Territory, Australia; University of St Andrews, UNITED KINGDOM

## Abstract

Beck’s theory suggests that forming negative self-cognitions is a key early step in the development of depression. However, others have suggested the reverse, arguing that depression leads to development of negative self-beliefs. As such, there is debate about whether these cognitions are precursors to, or alternatively are caused by, depression. Although Beck’s theory is supported in older adolescents, it has not been clearly seen in younger adolescents. This study aimed to assess the relation between two major self-cognitions (self-esteem and self-criticism) and depressive symptoms in early adolescence. Two-hundred and forty-three Australian adolescents (mean age = 12.08, 52% female) completed measures of self-esteem, self-criticism and depressive symptoms at baseline, then approximately 12- and 24-months later. Growth-curve modelling was used to assess changes in the variables. Cross-lagged analysis assessed whether either of the self-cognition variables predicted depressive symptoms, or if depressive symptoms predicted self-cognitions. Results indicated that self-criticism and depressive symptoms increased over the time period, while self-esteem decreased, and these changes were all related. Self-esteem predicted depressive symptoms from Time 2 to Time 3, while depressive symptoms predicted self-esteem from Time 1 to Time 2. Self-criticism did not predict depressive symptoms, nor did depressive symptoms predict self-criticism. These links appeared largely independent of gender. Self-esteem and depressive symptoms during the early adolescent period thus appear to have a somewhat reciprocal relation, while self-criticism does not appear to predict the development of depression. As such, while low self-esteem does appear to have an important role of in the development of depression in this age group, it is not strictly predictive, nor is this effect seen across all negative self-cognitions.

## Introduction

According to theory, negative self-beliefs are key precursors in the development of depression. Beck’s [1, 2] theory of depression—the ‘vulnerability’ model—posits that if an individual develops a negative conceptualisation of themself, they become more prone to depression. This cognitive style creates a vulnerability which is triggered when negative events occur. As such, the theory suggests that negative self-beliefs, along with negative beliefs about the environment and the future, are critical features in the development of depression. This is supported by evidence that individuals at high risk for depression display increased levels of negative self-beliefs [3]. However, other theorists have argued that depression leads to the development of negative self-cognitions [4–6]. The focus of the current research is to develop a better understanding of how self-cognitions relate to the development of depressive symptoms during the early adolescent period, and clarify temporal relations between negative self-beliefs and low mood.

With the dominance of two major conceptions of depression, Beck’s [1, 2] cognitive approach and Blatt’s [7] psychodynamic approach, two self-cognitions emerge as particularly relevant to depression: Self-esteem and self-criticism. Empirical research has largely focused on self-esteem, that is, the general attitude towards the self as a whole [8]. Beck [2] highlights self-esteem as particularly important because the overall tendency of an individual’s self-esteem, whether positive or negative, will direct their overall cognitive structure towards or away from depressive thinking.

Self-criticism is the punishment or derogation people deliver to themselves when they assess that they have not met internally instigated standards [9, 10]. As such, it is a broad pattern of thinking regarding the self that occurs in response to any perceived failure. Thus, like self-esteem it is a global self-cognition which theoretically should create a negative schema that forms the basis of depressive thinking [2].

The importance of self-cognitions in depression is argued to be especially relevant during adolescence. There is clear evidence that the incidence of depression increases as children progress through adolescence, with these differences demonstrated between the ages of 11 and 15 [11]. Furthermore, this increase is particularly apparent in girls [12, 13]. Theorists have long argued that a key goal of adolescence is to develop a cohesive self-concept [14–18]. Erikson [16] argued that throughout childhood, an individual collects different views of themself garnered through a variety of experiences but it is not until adolescence that they summarise these views as a cohesive identity. This information about self is garnered from a number of social sources, initially focused on parent input, but with increasing influence from peers as the developmental period progresses [19–21].

In line with this, Cole’s [22–24] model posits that, because of this developmental need to understand the self, negative self-beliefs have a particularly powerful effect on young people and thus are the pivotal component of depression in adolescents and children. However, other theorists argue that negative self-cognitions are not a precursor to depression. In contrast to Beck’s [1, 2] vulnerability model, the ‘scar’ model proposes that it is the experience of depressive symptomatology that leads individuals to experience reductions in their self-esteem, with depression causing individuals to interpret themselves more negatively [4–6]. In the ‘reciprocal-causality’ model, both pathways are present, such that self-esteem levels influence depression but depression also influences self-esteem [25]. Despite these alternatives, the vulnerability model has dominated the literature and has strong empirical support [26, 27], particularly in older adolescents and adults.

Given the preponderance of the vulnerability model, addressing negative self-beliefs is widely accepted as a core component of prevention of depression. Programs targeting adolescents use a wide range of techniques to improve and maintain high levels of self-beliefs. These include cognitive therapy and activities that foster coping abilities to reinforce a positive sense of self [28]. Other programs, following evidence that parent behaviours influence adolescent self-cognitions [21], focus on strengthening relationships between parents and children to prevent depression, while others provide school-level activities designed to improve self-concept across a range of areas [28]. However, the possibility that depressive symptoms may precede reduced self-worth suggests that these programs may be misdirected and potentially should be targeting other factors to prevent depression. As such, the question of whether self-cognitions predict depression, or the reverse, is vital when it comes to preventing depression in adolescents.

In support of the vulnerability model, longitudinal studies have found that self-esteem negatively predicts later depressive symptoms in adolescents. Lee and Hankin [29] found that, in a sample of 350 adolescents (mean age = 14.5 years), controlling for earlier depressive symptoms, self-esteem significantly negatively predicted depression five weeks later. Similar effects were found in a sample of 115 adolescents (mean age = 16.5 years) in whom self-esteem and depressive symptoms were measured 14 weeks apart [30], supporting the notion that self-esteem negatively predicts depression.

In research specifically comparing the vulnerability and scar models, Orth, Robins [26] Study 1 supported the vulnerability model in a sample of 2,403 adolescents, mean age 15.5 years at baseline. Assessed at three additional time-points, each two years apart, cross-lagged modelling examined three paths (Time 1 self-esteem–Time 2 depression scores, Time 2 self-esteem–Time 3 depression scores, Time 3 self-esteem–Time 4 depression scores). Three equivalent pathways from depressive symptoms to self-esteem were also assessed. All pathways from self-esteem were significant (β’s = -.09 to -.10, all *p*’s < .01), while no pathways from depressive symptoms to self-esteem were significant (all β’s = -.04, all *p*’s > .05). In slightly older populations, stronger support for vulnerability is found. In a similar design, Orth and colleagues’ [26] Study 2 (mean age = 18.3), found pathways from self-esteem to depression scores (β’s = -.20 to -.22, all *p*’s < .01). Replicating these findings, Rieger, Göllner [27], in a sample of 2,512 participants with mean age = 21.5, found similar pathways (β’s = -.23, -.24, *p*’s < .01). Both found minimal support for depressive symptoms to self-esteem pathways. As such, these studies provide strong empirical support for the vulnerability model.

Despite most evidence supporting Beck’s theory [1, 2], there have been some studies that have presented contradictory evidence. Shahar and Henrich [31] argued against Orth and colleagues’ [26] Study 1 conclusions, suggesting that although β < .10 (absolute value) may be statistically significant when sample sizes are very large, the practical meaning is questionable. Furthermore, the relation between self-cognitions and depression appears to differ depending upon participant age. Shahar and Henrich [31] divided a sample of 4,520 adolescents, who were tested at two points one year apart, into age groups. Cross-lagged modelling found, for those aged 14 to 16 at Time 1, that self-esteem did not predict depressive symptoms, nor vice versa, above the β = .10 level. However, for those aged 12 to 13 at Time 1, depressive symptoms predicted self-esteem (girls: β = -.17, *p* = .020, boys: β = -.19, *p* = .008), but not the reverse, supporting the scar model. In a sample of 110 adolescents (mean age 13.6 years) measured at baseline and after a 6–8 month interval, cross-lagged analysis suggested that self-esteem both predicted depressive symptoms (β = -.30, *p* < .05) and was predicted by depressive symptoms (β = -.22, *p* < .05), supporting the reciprocity model [32]. As such, while most research supports, to some extent, the vulnerability model regarding self-esteem and depression in older adolescents and young adults, this relation is not entirely clear in younger adolescents.

Research that has empirically explored the prospective links broadly between self-criticism and depression in adolescents has been mixed. In support of the vulnerability model Auerbach, Ho [33] examined a sample of 157 adolescents (mean age = 13.99 years), with five data collections over six months, finding that self-criticism predicted depressive symptoms. In a sample of 1,150 adolescents (mean age = 16.26), Cohen, Young [34] found that self-criticism predicted depressive symptoms over a six-month period, with measurements taken monthly, although the effect size was small (β = .06, *p* < .01).

Other research has found that self-criticism did not predict depressive symptoms, questioning the vulnerability model at least with regard to self-criticism. Little and Garber [35], using hierarchical regressions that controlled for sex, initial depression levels and concurrent levels of anger/aggression, examined a group of 486 5^th^ and 6^th^ graders (mean age = 11.4 years). Self-criticism did not significantly predict depression scores three months later. Kopala-Sibley, Zuroff [36] sampled 241 adolescents (mean age = 12.57) who were tested at baseline, then two years later. In a structural equation model controlling for dependency, life events and anxiety, Time 1 self-criticism did not predict Time 2 depressive symptoms. Adams, Abela [37] followed 56 children (mean age = 10.6 years) over six weeks and found that self-criticism alone did not significantly predicted depressive symptoms. Similarly, Abela and Taylor [38] examined 303 3^rd^ and 7^th^ graders, using a hierarchical regression that contained both self-criticism and self-esteem, plus negative events, and found that after a six-week delay, self-criticism individually did not predict depression scores in either group. However, upon examination of interaction effects, they found that in 3^rd^ grade boys and girls and 7^th^ grade boys who had both low self-esteem and high self-criticism, negative events predicted depression scores, suggesting a more complex relation between self-criticism and depression.

Shahar, Blatt [25] compared the vulnerability and scar models, examining self-criticism and depressive symptoms in a two time-point cross-lagged study of 6^th^ and 7^th^ graders, aged 11–14 years. Four hundred and sixty students (50% girls) completed measures at baseline and 12 months, with no significant relations between the two variables found for boys. However, for girls, both pathways were significant, with self-criticism predicting depressive symptoms and depressive symptoms predicting self-criticism. Thus, reciprocal causality was seen, but only for girls.

### Omissions in the literature

Few previous studies have included both self-esteem and self-criticism in the measurement models. One exception, Abela and Taylor [38], did include both variables, however, only examined changes over a brief period (six weeks). Given the changes that are expected across the early adolescent period as evidenced by previous research [12], longer term research is required to full explore the nature of these relations in this age group.

In contrast to older age groups [26, 27], there is limited understanding of the relations between self-esteem or self-criticism to depression in the early adolescent period. This is particularly pertinent, given that it is this age group in which cognitive factors that predispose individuals to depression are expected to be developing, prior to the increase in symptomatology seen in older adolescents [11]. As such, to fully understand whether Beck’s [1, 2] vulnerability model is supported in this key developmental period, more research is needed in this population.

As such, our understanding of the precise relation between the two major self-cognitions and depression remains limited. These constructs are conceptually closely linked, and thus difficult to empirically disentangle. Nevertheless, Beck’s [2] model suggests that self-esteem and self-criticism are separate constructs that both directly increase vulnerability to depression. Other theories support this, suggesting that all self-cognitions, generally, have a similar relation with depression [39]. Empirical data suggest that although the two variables are strongly negatively correlated, around *r* = —.5 [21], they do not appear to be perfectly orthogonal constructs. There is also suggestion of a more complex relation between the various self-cognitions, which may have flow-on effects on depression. For example, Katz and Nelson [40] propose that self-esteem precedes self-criticism, whereby self-criticism may develop following a reduction in self-esteem. This suggests that self-esteem may have indirect effects on depression, via its influence on self-criticism, in addition to possible direct effects. Alternatively, given that self-esteem is the broad valuation of the self as a whole, it is also possible that all other self-cognitions, including self-criticism are, to some extent, subsumed by self-esteem. As such, it is possible that any relation demonstrated between self-criticism and depression is merely a function of the relation between self-esteem and depression. The relation between self-esteem and self-criticism remains unclear. Thus, to develop an in-depth understanding of how self-esteem and self-criticism relate to depression, research is needed which models relations between all three variables.

Little research has examined the changes over time in these variables during adolescence, in relation to each other. While it appears clear that depression increases during adolescence [11] and self-esteem decreases [41]–although some research has suggested a less linear change in self-esteem [42]–the trajectory of self-criticism is unclear. Given the theoretical links [2] between self-criticism and depression, it is reasonable to expect that, like depression, self-criticism increases during this period. Likewise, based on theory it would be expected that these changes are connected. However, to date this has not been tested empirically. This study is thus the first to examine how changes in adolescent self-criticism, self-esteem and depressive symptoms in relation to each other. It is also one of few studies to examine whether self-criticism and self-esteem predict depressive symptoms, controlling for the effects of each.

### The current research

This is the first extended longitudinal study of a non-clinical sample to assess self-criticism and self-esteem during early adolescence, a developmental period in which depression rates are expected to increase. The aim of the current research was broadly to examine how the development of self-criticism and self-esteem relates and contributes to the development of depression in early adolescence. The research focused on addressing the following questions:

1 Do self-criticism levels in adolescents change over time (ages 12 to 14)? If so, is this change related to changes in self-esteem and depressive symptoms?

If these are answered in the affirmative, it will be an indication that further analysis is appropriate and thus the following questions will be addressed:

2 Do self-criticism and self-esteem predict depressive symptoms (vulnerability model) or is the reverse seen (scar model) during early adolescence? Further, how do self-esteem and self-criticism relate to each other, with regard to depressive symptoms?

As some research has demonstrated gender effects [25], we also intended to test for this possibility. To address these aims we measured self-criticism, self-esteem and depressive symptoms in a community sample of 7^th^ graders (Time 1; T1), again at approximately 12 months (Time 2; T2) and again at approximately 24 months from baseline (Time 3; T3).

Regarding Question 1, we hypothesised that all three variables would display significant linear change over the testing period (self-criticism and depressive symptoms increase and self-esteem decrease) [11, 41]. Based on theoretical assumptions [2], we also expected that all three changes would be correlated (self-criticism increase would positively relate to depressive symptom increase and negatively to self-esteem decrease, and self-esteem decrease would negatively relate to depressive symptom increase).

Regarding Question 2, we made hypotheses in line with the vulnerability model. Although there has been some dissent regarding the empirical veracity of this model [31], theoretical models guided by the vulnerability hypothesis remain dominant [2, 43] and the empirical evidence weighs most strongly towards vulnerability [26, 27]. As such, we expected that self-esteem would negatively predict depressive symptoms from T1 to T2 and T2 to T3, but depressive symptoms would not predict self-esteem from T1 to T2 or T2 to T3. Given the mixed findings for self-criticism and similar dominance of the vulnerability model in this area, we also used this model to guide hypotheses for self-criticism. As such, we expected that self-criticism would positively predict depressive symptoms from T1 to T2 and T2 to T3, but depressive symptoms would not predict self-criticism from T1 to T2 or T2 to T3. We expected, based on evidence that depression is more prevalent in girls than boys in this age group [12, 13], that higher levels of the two self-cognitions and depressive symptoms would be seen for girls compared to boys. Given the lack of previous empirical evidence, we did not make specific predictions regarding the relations between self-criticism and self-esteem. These research aims were largely met using the following methodology.

## Method

### Design

To examine the longitudinal relations between adolescent self-esteem, self-criticism and depressive symptoms these three variables were measured at three time points in a sample of students who were initially in Grade 7. To examine change in these variables over time (question 1), growth curved modelling was employed. To examine the predictive relations between the variables, controlling for the effects of the variables (question 2), cross-lagged modelling was used. Gender was also added as a control variable to both models.

### Participants

Two-hundred and forty-three (52% female) Grade 7 students participated at T1. Mean age was 12.08 (*S*.*D*. = 0.43), range 11–13 years. With approximately 685 eligible students across all school, this represented an uptake rate of around 35%. At T2, 245 (50% female) students participated, and at T3, 219 (51% female) participated. This represented a drop-out rate of 10% from T1 to T3. This primarily occurred due to non-attendance at school on testing days, and did not appear to be related to any specific demographic factors. At T1, 82% of participants were Australian born, 7% born in Europe, 6% born in Asia and 4% were North American born. Fifty-four percent reported having two Australian-born parents, 26% reported having one parent born outside Australia and 20% reported having two parents born outside Australia. See Table 1 for further demographic details.

**Table 1:** Demographic information (Time 1).

**Overseas born parents (n = 160)**	
Europe	71 (44%)
Asia	35 (22%)
North America	15 (9%)
New Zealand/Pacific	14 (9%)
Middle East	11 (7%)
South America	9 (6%)
Africa	5 (3%)
**Parent education (n = 242)**	
Both completed university degree	163 (67%)
One completed university degree	51 (21%)
Both completed high school (only)	10 (4%)
One completed high school (only)	15 (6%)
Neither completed high school	3 (1%)
**Living situation (N = 243)**	
Both parents	215 (88%)
Dividing time between two parents’ homes	11 (5%)
One parent (only)	17 (7%)

### Recruitment

In 2013 participants were recruited from six independent schools (defined as schools not administered by either the state government or Catholic education systems) across the greater Sydney area after approximately 30 schools were approached. Although all schools were fee-paying, Index of Socio-Cultural Educational Advantage scores indicate that they did not significantly deviate from other schools in Australia. Participating schools allowed the researchers to advertise the study via email, newsletter announcements and school presentations, and distributed information and consent forms to all Grade 7 students. To reduce potential response bias, researchers encouraged school staff to collect forms from all eligible students. Consent forms contained options to either consent or refuse consent. Only those who provided written consent from both student and a parent/guardian were included in the study. No inducement to participate was provided beyond a broad report for schools describing the overall scores of their student group.

### Procedure

Participants completed a battery of measures at three time-points, each approximately 12 months apart (2014, 2015, 2016). The battery contained self-report measures and demographic information, including self-identified gender. This testing was part of a larger data collection, with other findings regarding relations between parenting and child self-cognitions reported elsewhere [authors’ citation]. Parents were also invited to complete measures (not reported here). Testing sessions were conducted at each participating school by the researchers and/or school counselling staff. Students were seated individually, with measures administered via computer or paper-and-pen, and testing was completed within approximately 30 minutes. Participants could withdraw from the research at any time. Students who scored in the very elevated range for depressive symptoms were identified to school counsellors. All data were then de-identified prior to analysis. This study, entitled ‘The Self-Esteem and Depression in Adolescents (SEDA) Study’, was approved by The University of Sydney Human Research Ethics Committee, approval number 2013/682.

### Measures

#### Self-criticism

Levels of Self-Criticism Scale–Internalised Self-Criticism subscale [9]. This subscale of the LOSC was used to assess self-criticism that arose from not meeting internalised standards, with high scores indicating greater self-criticism. It contains 10 items on a seven-point Likert scale. The measure has been used with adolescents [44] and found to have good convergent validity with Depressive Experiences Questionnaire- Self-Criticism [7] and reliability [9]. The LOSC demonstrated good internal consistency in the current study (α’s T1, T2, T3, respectively = .88, .88, .89). Questions include, “I feel like a failure when I don’t do as well as I would like”.

#### Self-esteem

Rosenberg Self Esteem Scale [45]. The RSE is a widely used measure with adolescents [26, 46, 47] that assesses global self-esteem. There are 10 items (scores range 1 to 4); high scores indicate high self-esteem. It displays good reliability [48], and good construct validity in adolescents when relations are examined with current life experiences [49]. The RSE demonstrated good internal consistency in the current study (α’s T1, T2, T3, respectively = .87, .89, .91). Questions include, “On the whole, I am satisfied with myself”.

#### Depressive symptoms

Children’s Depression Inventory-2 Self-Report Short [50]. CDI-2 is an updated version of the original measure developed by Kovacs [51] to assess depressive symptomology in children aged 7 to 17 years. The CDI is the most widely used self-report measure for depression in young people and has been shown to display good validity when compared to a structured interview for depression [52]. In the CDI-2 Short Form, depressive symptoms are measured via 12 questions (scores range 0 to 3), with higher scores indicating more depressive symptoms. Scores of ≥ 7 for girls aged 7–12, ≥ 12 for girls aged 13–17 or ≥ 9 for boys aged 7–17 are consider ‘very elevated’. The CDI-2 demonstrated good internal consistency in the current study (α’s T1, T2, T3, respectively = .84, .83, .83).

### Statistical analyses

All structural equation modelling was conducted using SPSS Amos 22. To address Question 1, growth curve modelling was applied using observed variables of total scores for self-criticism, self-esteem and depressive symptoms at T1, T2 and T3. Latent variables for intercept (indicating mean score on the measure at T1) and slope (indicating the rate of change in the measure across T1, T2 and T3) were assessed for each of the three constructs, with regressions to the intercept variable constrained to 1 and regressions to the slope intercepts constrained to 0, 1 and 2 for T1, T2 and T3 (to specify linear change over time), respectively. Covariances between all the latent variables were estimated to determine whether changes in the measures were related to each other. Gender as a time-invariant predictor was then added to the model, with regressions from gender to all intercept and slope variables, to assess whether scores at T1 and rate of change over time were different for girls compared to boys. Residuals were estimated for all endogenous variables.

To address Question 2, cross-lagged modelling was employed. This involved observed variables of the total scores of all three variables at all three time-points in a single model. Regressions between the same construct from T1 to T2 and T2 to T3 were included to control for autoregressive effects. To examine predicted relations between the three constructs, regressions were included from each variable at T1 to the other two variables at T2, and the same from T2 to T3. Covariances between the three variables at T1 were estimated, and similarly variables at T2 and T3. Gender was added to the model, with regression lines from gender to all observed variables, to assess whether mean scores of the variables differed for girls and boys, controlling for autoregressive effects.

The difficulties with determining a meaningful minimum sample size appropriate for Structural Equation Modelling (SEM) have been well noted [53]. As Barrett [54] points out, the value in conducting power analyses at all in the context of SEM is questionable. However, given the relative simplicity of the proposed models, and the assertion that, in general, samples of greater than 200 participants are needed [54], it is likely that a sample of 250 participants will provide adequate power for the current study.

In line with previous research in this area [55], fit indices selected to assess all models were the Comparative Fit Index (CFI), the Tucker–Lewis Index (TLI) and the Root Mean Square Error of Approximation (RMSEA), in addition to χ^2^. For CFI and TLI > .95 demonstrates good fit and > .90 demonstrates adequate fit, and for RMSEA < .05 demonstrates good fit, while < .08 demonstrates adequate fit [56, 57].

### Data preparation

The data were assessed using guidelines developed by Kline [53] to confirm appropriateness for SEM analysis. They were found to be non-singular with eigenvalues > 0 and no extreme collinearity was found, as demonstrated by *R*^*2*^ < .90 when each variable in the model was regressed on all the other variables. No outliers were observed when assessed as per guidelines [58]. All variables met assumptions of normality, and therefore no transformations were conducted. Testing indicated that it was appropriate to replace missing data. Little’s MCAR test indicated that all data were missing completely at random, except T1 self-esteem and T1 self-criticism. However, these had <2% missing values, which is considered negligible [59]. Thus Relative Mean Substitution [60] and Maximum Likelihood (ML) procedures in AMOS were implemented.

To test for clustering effects within school groups, intra-cluster correlation co-efficients (ICCs) were assessed. One-way ANOVAs were conducted on Time 1 outcome variables (self-esteem, self-criticism and depressive symptoms). These analyses indicated no significant effect of group on the outcome variables (all *p’*s > .111). ICCs were assessed using the following formula:

$$ICC\;(\rho )=\frac{s_{b}^{2}}{\left(s_{b}^{2}+s_{w}^{2}\right)}$$

where *s*_*b*_^*2*^ = between cluster variance, and *s*_*w*_^*2*^ = between cluster variance. This analysis demonstrated low ICCs for the variables (ρ’s = .020, .022, .037, for self-esteem, self-criticism and depressive symptoms, respectively), suggesting low correlation between responses within the school groups. As such, it was appropriate to pool the data from the six schools.

## Results

### Descriptive statistics and correlations

See Table 2 for correlations, means, standard deviations and ranges. All variables were significantly correlated at the 0.01 level. As per Hinkle et al.’s [61] rule of thumb, .7 to .9 indicates high correlation, .5 to .7 indicates moderate correlation, .3 to .5 indicates low correlation, and less than .3 displays little correlation. Correlations from T1 to T2 and T2 to T3 of the same variable were moderately correlated (.51 to .70). Similarly, correlations between self-esteem and depressive symptoms, from T1 to T2 and T2 to T3, and the same periods from depressive symptoms to self-esteem were moderate (-.53 to -.57). However, relations between these two variables and self-criticism during the same time periods were notably lower, in the little to low correlation range (self-criticism/depression score: .22 to .37; self-criticism/self-esteem: -.28 to -.37).

**Table 2:** Descriptive statistics and Pearson’s correlations.

	Self-Criticism T1	Self-Esteem T1	Depression T1	Self-Criticism T2	Self-Esteem T2	Depression T2	Self-Criticism T3	Self-Esteem T3	Depression T3
**Correlations**									
T1 Self-Criticism	-								
T1 Self-Esteem	-.44**	-							
T1 Depression	.42**	-.71**	-						
T2 Self-Criticism	.51**	-.31**	.29**	-					
T2 Self-Esteem	-.35**	.62**	-.57**	-.42**	-				
T2 Depression	.37**	-.53**	.70**	.45**	-.76**	-			
T3 Self-Criticism	.46**	-.31**	.23**	.56**	-.37**	.33**	-		
T3 Self-Esteem	-.30**	.51**	-.42**	-.28**	.66**	-.53**	-.42**	-	
T3 Depression	.25**	-.43**	.47**	.22**	-.56**	.56**	.37**	-.76**	-
**M (S.D.)**									
All	35.07 (12.85)	30.54 (5.06)	4.49 (4.02)	38.61 (12.27)	30.28 (5.36)	5.04 (4.02)	40.85 (12.1)	29.79 (5.66)	5.15 (4.01)
Girls	36.32 (13.37)	30.78 (5.19)	4.22 (4.03)	39.85 (12.81)	29.48 (5.05)	5.47 (3.87)	42.83 (11.44)	29.02 (5.17)	5.63 (3.98)
Boys	33.72 (12.17)	30.28 (4.91)	4.78 (4.01)	37.36 (11.61)	31.07 (5.56)	4.6 (4.13)	38.79 (12.48)	30.58 (6.05)	4.65 (4.00)
**Range**	10–70	15–40	0–20	10–70	13–40	0–22	14–68	10–40	0–22
**N**	243	243	243	245	245	244	218	217	218

** p < .01

### Growth curve models

**Fig 1:**
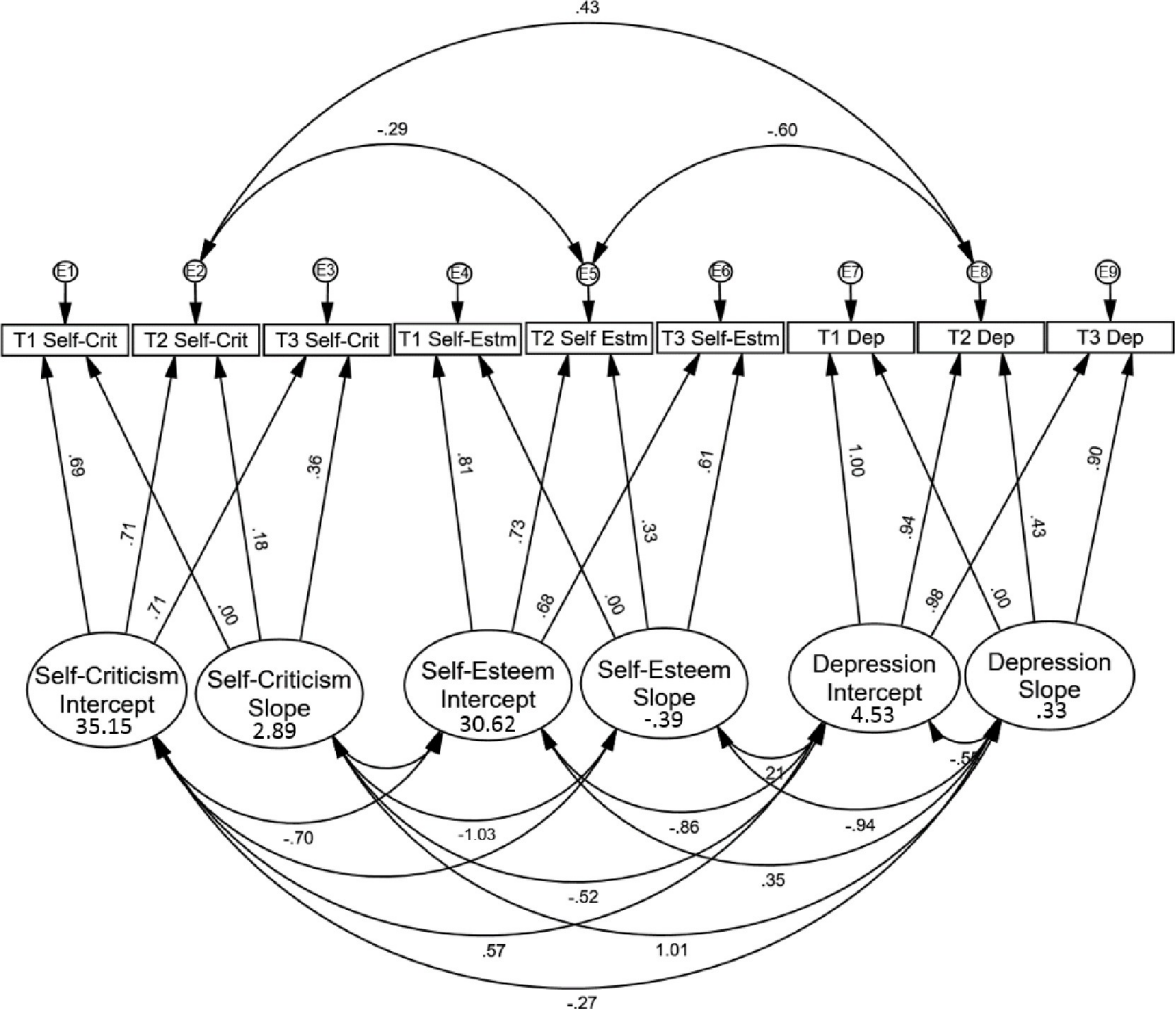
Growth curve model. Standardised estimates are displayed. All statistics displayed are significant at p < .05. Estimates for the latent variables appear inside the ovals. Estimates for covariances appear above or below curved, double-headed arrows. T1 = Time 1; T2 = Time 2; T3 = Time 3; Self-Crit = Self-Criticism; Self-Estm = Self-Esteem; Dep = Depressive symptoms; E = residual.

The planned model was constructed and initially only complete cases were used to enable use of modification indices (MI) to improve model fit, as MI cannot be implemented in AMOS with ML [62]. Covariances seen in Fig 1 reflect changes that best improved model fit, as indicated by modification indices, with all modifications guided by theoretically justified relations, as per recommendations [53, 62]. Once this identification of the model was complete, the full dataset was analysed. Model fit was found to be good: χ^2^ (17, *N* = 292) = 20.199, *p* = .264, CFI = .997, TLI = .993, RMSEA = .025.

#### Estimates

Estimates for the latent variables appear in Fig 1. Notably, self-criticism significantly increased over time (estimate = 2.89, *S*.*E*. = .42, *p* < .001), self-esteem decreased (estimate = -.39, *S*.*E*. = .17, *p* = .027) and depressive symptoms increased (estimate = .33, *S*.*E*. = .13, *p* = .013). Estimates for covariances between latent variables also appear in Fig 1. The self-criticism and depressive symptom slopes were significantly correlated (4.01, *S*.*E*. = .90, *p* < .001), as were the self-criticism and self-esteem slopes (-4.05, *S*.*E*. = 1.13, *p* < .001) and self-esteem and depressive symptom slopes (-3.08, *S*.*E*. = .40, *p* < .001). This suggested that changes in all three variables were related to changes in the other variables.

#### Gender effects

Gender (coded boys = 0, girls = 1) was added to the model. Initial fit was good: χ^2^ (20, *N* = 292) = 26.409, *p* = .154, CFI = .995, TLI = .985, RMSEA = .033. However, when non-significant regressions from gender were sequentially removed from the model (i.e. regression with highest p-value removed first), until only significant regressions remained, a better fit was found: χ^2^ (23, *N* = 292) = 27.787, *p* = .224, CFI = .996, TLI = .990, RMSEA = .027. This more parsimonious model was assessed and included three significant regression paths from gender to the latent variables. Girls’ self-esteem reduced at a significantly greater rate than boys’ (β = -.24, *p* = .006), and girls’ depressive symptoms increased at significantly greater rate than boys’ (β = .15, *p* = .014). Additionally, self-criticism was significantly higher for girls at T1 (β = .16, *p* = .012). In this model, the self-esteem and depression score simple slopes were no longer significant (*p*’s = .067, .180, respectively), with boys scores remaining relatively stable over time. However, the self-criticism simple slope remained significant (2.90, *S*.*E*. = .42, *p* < .001), with both boys’ and girls’ increasing in self-criticism. The intercepts remained significant (all *p*’s < .001), and the correlations between all three simple slopes demonstrated in the initial model remained significant (all *p*’s < .001).

### Cross-lagged models

**Fig 2:**
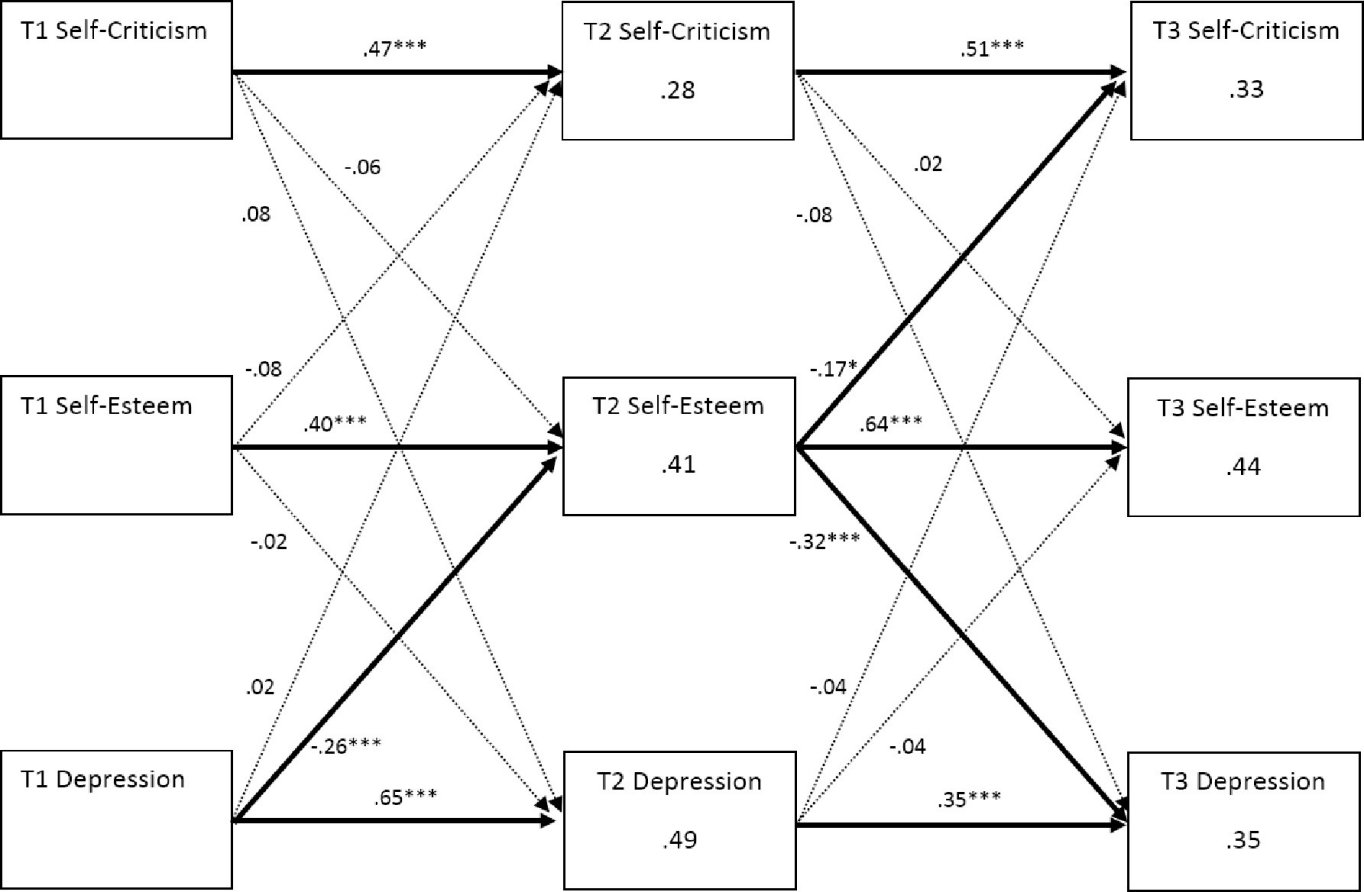
Cross-lagged model. Standardised estimates are displayed. Dotted lines indicate non-significant pathways. Covariances and error terms have been excluded from figure for clarity. T1 = Time 1; T2 = Time 2; T3 = Time 3. * p < .05; *** p < .001.

In the initial model, although χ^2^ was significant, (9, *N* = 292) = 18.793, *p* = .027, other indices indicated adequate fit: CFI = .992, TLI = .958, RMSEA = .061. See Fig 2 for model.

#### Estimates

Estimates for regression weights are displayed in Fig 2. As expected, T2 self-esteem predicted T3 depressive symptoms (β = -.32, *p* < .001), however, T1 self-esteem did not predict T2 depressive symptoms (*p* = .748). Furthermore, T1 depressive symptoms predicted T2 self-esteem (β = -.26, *p* < .001) although T2 depressive symptoms did not predict T3 self-esteem (*p* = .590). Self-criticism did not predict depressive symptoms or self-esteem at any point, nor did depressive symptoms predict self-criticism, although T2 self-esteem did predict T3 self-criticism (β = -.17, *p* = .045).

#### Gender effects

Gender was added to this model to assess whether it influenced these results, with regression pathways from gender to all observed variables. Model fit remained similar, but when non-significant pathways from gender were removed sequentially, model fit was improved: χ^2^ (15, *N* = 292) = 23.114, *p* = .082, CFI = .993, TLI = .975, RMSEA = .043 and this more parsimonious model was retained. Three pathways from gender were significant: gender to T1 self-criticism (β = .13, *p* = .016), suggesting that girls’ self-criticism was significantly higher than boys’ at T1, gender to T2 self-esteem (β = -.14, *p* = .002), suggesting that girls’ self-esteem was significantly lower than boys’ at T2 and gender to T2 depression scores (β = .09, *p* = .030), suggesting that girls’ depressive symptoms were significantly higher than boys’ at T2. Regressions from T2 self-esteem to T3 depressive symptoms and T1 depressive symptoms to T2 self-esteem remained significant (*p*’s < .001), as did the regression from T2 self-esteem to T3 self-criticism (*p* = .046).

#### Post-hoc analysis (removing self-esteem)

Given the unexpected finding that self-criticism did not predict depressive symptoms, we tested the possibility that relations may be present if self-esteem was removed from the model. This enabled assessment of possibility that relations demonstrated between self-criticism and depression in other literature may have reflected the influence of self-esteem, rather than self-criticism per se. As the model including gender was a better fit, it was retained and pathways to and from self-esteem to depressive symptoms and self-criticism were removed. Model fit was reduced: χ^2^ (23, *N* = 292) = 67.301, *p* = .000, CFI = .962, TLI = .910, RMSEA = .081. Further, all pathways from self-criticism to depressive symptoms and from depressive symptoms to self-criticism remained non-significant (all *p*’s > .149).

## Discussion

Both depressive symptoms and self-criticism significantly increased from ages 12 to 14, while self-esteem significantly decreased, as demonstrated by growth curve analysis. These changes were also significantly associated with each other. Furthermore, initially depressive symptoms predicted reduction in self-esteem, but later, lower self-esteem levels predicted increased depressive symptoms, shown through cross-lagged analysis. Self-criticism did not significantly predict either depressive symptoms or self-esteem, although self-esteem did predict reduced self-criticism from ages 13 to 14. This pattern remained unchanged when controlling for gender.

These findings ultimately support the possibility of a reciprocal-causality model in this population. Although pathways from self-esteem to depressive symptoms and the reverse for both time-lapses were not seen, there was nevertheless a sense of reciprocity seen across the two-year period. Thus, depressed mood appears to influence how adolescents evaluate their own worth, and negative beliefs about the self as a whole appears to increase the likelihood of developing depressed mood, at least for this age group.

The findings did not suggest an indirect relation in which one self-cognition predicted the other, which subsequently predicted depressive symptoms. There was evidence, in support of Katz and Nelson’s [40] assertion, that reduced self-esteem predicted subsequent self-criticism, demonstrated here from Time 2 to Time 3. However, there is no suggestion in the current data that self-criticism predicts later depressive symptoms. Further, even when self-esteem was removed from the model, self-criticism did not predict depressive symptoms. As such, based on these data, it is unlikely that there is any significant direct predictive relation between self-criticism and symptoms of depression.

The evidence for some reciprocity between self-esteem and depressive symptoms rather than vulnerability may reflect the age of the current sample. Much of the research which has supported the vulnerability model over the scar model has been on older adolescents (15 years) and adults [26, 27]. Shahar and Henrich [31] supported the scar model but only in a young adolescent sample, aged 12 to 13. Similarly, Burwell and Shirk [32], in a sample with a mean age of 13.6, demonstrated reciprocity between self-esteem and depressive symptoms. Shahar and Henrich [31] argued that, in line with developmental theory [15, 16], self-beliefs are more changeable during this period, thus they are more susceptible to influence from factors such as depressed mood.

These results suggest a refinement of Beck’s [1, 2] theory that all types of negative beliefs about the self generally create vulnerability to depression. At least for early adolescents, self-esteem appears to be more important than self-criticism in the development of depression. Although changes in self-criticism and depressive symptoms appear associated, these variables do not specifically predict each other. Our findings are in line with previous research, which has failed to demonstrate prospective links between self-criticism alone and depression [35, 37, 38, 42].

These findings may speak to the conceptual differences between the two self-cognition constructs. Self-esteem is considered a general attitude towards the self as a holistic object [8]. In contrast, self-criticism is a general approach to the self as a whole, but is contingent upon self-perceived failure [9]. While Beck [2] specified that negative life events are necessary in combination with negative self-cognitions to increase risk for depression, this may be more true for self-criticism–a thinking style that may lay dormant unless activated by a negative event–than for self-esteem–a more ongoing sense of self rather than response to events. There is some empirical evidence to suggest that high self-criticism alone is not enough to confer increased vulnerability to depression and that negative life events are also necessary. Abela and Taylor [38] found that while self-criticism alone did not predict depression scores, a significant interaction effect between self-criticism, self-esteem and negative life events was present, such that when participants with higher levels of self-criticism and lower levels of self-esteem experienced a negative life event, this combination significantly predicted depressive symptoms.

### Gender effects

These analyses also demonstrated some support for our expectation of higher levels of negative self-cognitions and depressive symptoms for girls compared to boys, with some significant gender differences seen. Notably, girls’ depressive symptoms increased, and self-esteem decreased, at a significantly greater rate than boys’ symptoms. At the start of the test period (age 12), boys and girls demonstrated similar levels of self-esteem and of depressive symptoms. However, at 13 years, controlling for initial levels of the variables, girls’ self-esteem was significantly lower, and depressive symptoms significantly higher, than boys’ symptoms. At 14 years, controlling for autoregressive effects, there was no significant difference in depressive symptoms or self-esteem levels for girls and boys, suggesting the possibility that the peak point of differentiation of these variables is at around age 13. As such, these findings provide some support for the prediction that negative symptoms and cognitions would be higher in girls than boys

However, some other aspects of the findings provide minimal support for gender differences. When overall models were examined, the major relations demonstrated between the three variables remained unchanged when gender was added. In the growth curve model, the changes in the variables remained significantly related. Similarly, in the cross-lagged model, Time 1 depressive symptoms continued to predict Time 2 self-esteem and Time 2 self-esteem continued to predict Time 3 depression scores, while self-criticism continued not to predict depressive symptoms or self-esteem. Taken together, our findings suggest some evidence for higher levels of depressive symptoms, and higher rate of increase of these symptoms, in girls. However, no evidence of difference in the overall pattern of relations between self-esteem, self-criticism and depression for girls compared to boys was demonstrated.

### Clinical implications

The current study examined relations between self-cognitions and depressive symptoms in a sample of healthy adolescents. Nevertheless, the finding that low self-esteem, but not high self-criticism, conveys direct risk for depressive symptoms may have important implications for the treatment of adolescents with, or at risk of, depression. It supports targeting self-esteem in interventions that aim to prevent depression, but also suggests that self-criticism should not necessarily be a specific focus. Further, the evidence of a reciprocal relation between self-esteem and depressive symptoms may have ramifications for depression treatment. Clinicians such as Greenberger and Padesky [63] have recommended focusing on ‘hot’ cognitions–those that are the most emotionally salient to the client–when conducting cognitive therapy. These findings suggest that, in addition to this approach, it may also be particularly important to ensure cognitions relating to negative self-esteem are also addressed, regardless of whether they are especially emotive to the client, as part of relapse prevention.

### Strengths and limitations

These results should be interpreted in light of the following limitations. The observational nature of these data limits the causal assumptions that can be drawn from this research. Although cross-lagged models are designed to address this issue by controlling for autoregressive effects, nevertheless causal relations cannot be definitively determined. Furthermore, although depressive symptoms were measured, it is unclear whether these results would generalise to clinically diagnosed depression. Other factors that have been found to influence relations between self-cognitions and depression, such as negative events [38], were not included in the current research. While this enabled focus on the primary variables of interest, this is an area of research which would further elaborate on these relations.

All participants were the same grade, and most were the same age at each time point. However, age was not measured in months which would have provided more variation, and therefore the analysis may have been improved by controlling age in months. Nevertheless, given that the age range would have remained limited, this is unlikely to have had a major effect. Cross-lagged modelling is an analytical approach that is widely used and well accepted in the field (e.g. [64, 65]). In recent years, some researchers have argued that it does not take into account the possibility of stable between-person differences in the variables, and therefore runs the risk of amalgamating between-person effects with within-person effects [66, 67]. As such, this is a potential limitation of the study, which may lead to an over-estimate of the effects, and should be considered when interpreting these findings. Nevertheless, the general pattern of findings is likely to be unchanged with an alternative cross-lagged analytical approach.

This research also has a number of strengths. It is one of few studies to examine self-criticism and self-esteem together in relation to depressive symptoms and, as such, is one of the first studies to uncover the links between these three constructs. Furthermore, by using both growth curve and cross-lagged approaches, we were able to address two related but separate issues: whether the change in these variables is related and whether they predict each other. By using three data-points rather than two, we were able to examine the repeated relations and thus develop a more comprehensive understanding of these connections.

## Conclusion

Overall, these findings suggest that, from ages 12 to 14, self-esteem appears closely connected to depressive symptoms, however, the relation is somewhat reciprocal rather than strictly predictive. In contrast, self-criticism, although related to depressive symptoms, does not directly predict them, nor is it predicted by depressive symptoms. Initially, depressive symptoms predict lower self-esteem, but later low self-esteem predicts greater depressive symptoms. As such, the results suggest that the vulnerability model or scar model alone are not enough to explain the relation between these variables in young adolescents. The vulnerability model may apply during late adolescence and early adulthood when self-cognitions become more crystallised. However, in the early adolescent years while these cognitions are still developing, there appears to be a more reciprocal relation between self-esteem and depression, with each influencing the other.
